# The Nursing Library

**Published:** 1921-01-15

**Authors:** 


### THE NURSING LIBRARY.

**The Child Welfare Movement.** By Janet E. Lane-Claypon, M.D., D.Sc., Dean of the Household and Social Science Department, King's College for Women, University of London. Late Medical Inspector of Child Welfare under the Local Government Board. (G. Bell and Sons, Ltd. Price 7s. net.)

Miss Lane-Claypon's book is of the kind which steps at once into the library of the social worker as indispensable. Years of experience in the inspection of Child Welfare Centres have shown her the strength of the movement, its weak points, and the best lines on which to develop it. The style is clear, and the information is so pleasantly imparted as to be a source of great interest to the general reader, as well as to all health visitors, nurses, and municipal authorities. A wealth of instruction in the organisation of welfare centres is afforded, and valuable forms, extracts from acts dealing with the matters in hand, of reports, schemes and statistics, are furnished in the Appendices. Unquestionably a book for the institution library and for all engaged in child-welfare work. It comprises within the compass of 321 pages a complete guide to the subject, and is provided with a good index

**Letters of an Australian Army Sister.** By Annie Donnell of the Third Australian General Hospital. (Sydney : Angus Robertson, Ltd. Price 6s. net.)

Very pleasantly written, with evident marks of the gift of a good raconteur, the letters of Sister Donnell were too heavily handicapped at the outset by the censor to give her a fair chance. In particular, the reader expecting to hear something of the Gallipoli campaign from one who witnessed many stirring events is doomed to disappointment. But as the series advances the letters grow less reticent. The book is indispensable to those who desire to capture the real thrill of constant bombardment from the air. We have read nothing to compare with the presentment of the nurses' terrible trials and experiences in France during the autumn and winter of 1917-1918 in this particular. In another way, in the charm with which it gives the subtle flavour of Australian patriotism, the sort which flowed out bountifully to the old country, yet held a deeper sentiment reserved exclusively for the "Aussies," the book is a success in a genre very difficult of achievement.

**Surgical Operations.** A Text-Book for Nurses. By E. W. Hey Groves, M.D., B.Sc., M.S., F.R.C.S. (London : Henry Frowde, and Hodder & Stoughton. Price 21s. net.)

Preceded by few others in the same category, this superior work upon modern practical surgery and surgical technique, in relation to the nursing aspect, should find its way into the hands of every surgical nurse. Although written for the benefit of nurses in training, its value will be more appreciated by the qualified nurse when following up a career as surgical sister or charge of the operating theatre. In such capacities it is absolutely essential that her knowledge of operative procedure and technique, throughout the varying manifestations, is automatic and complete. This knowledge can only be acquired by experience, aided by the possession of the true surgical instinct, and so eventually evolve her into the perfect assistant at the side of the operation table. Text-books as a rule afford but little help, being limited in information; the catalogues of surgical-instrument makers are a common channel through which the necessary up-to-date knowledge is obtained regarding the uses and varying patterns of instruments required for varying types of operations. Thus the real "want" of a reference guide existed, and the author has foreseen and supplied the want in his volume. Therein is contained a veritable open sesame of practical teaching upon every phase and process connected with operative work coming within the nurse's province, upon whose accuracy and prompt skill depends so much of the surgeon's success. The simple essentials are explained with the same care and detail as the larger and more involved. For each type of case every requirement is tabulated in order—sutures and needles; posture and position of patient; the reason of the operation explained; and, what is of utmost value, emergency points are indicated, passing on to special points in after-care and treatment. Special chapters are devoted to the modern attainment of asepsis in every department and branch, and a useful catalogue of instruments is included. Throughout the book is generously and elaborately illustrated, making the reading a most interesting and pleasurable study. As a reference book it should prove invaluable to the surgical nurse, and be regarded as an important acquisition to both the private and institutional library.

**Internal Medicine for Nurses.** Third Edition. By C. B. Farr, A.M., M.D. With 70 engravings and 6 plates. (Philadelphia and New York: Lea & Febiger, 1920.) 408 pp.

Dr. Farr's work, "Outlines of Internal Medicine," should be useful as a book of reference for junior medical students, but it is too complex and too lacking in all nursing technique to be of value as a practical text-book for nurses; it is evident the author is in the habit of lecturing to students; he writes mainly in the interests of the embryo physician, and ignores the aspect of medical diseases from the standpoint of the nurse.

The work is divided into ten parts; unfortunately the classification is so arranged that in many instances the text is repetitional, and the author fails to maintain throughout the high standard of clear and lucid explanation which he attains in his descriptions of the making of a blood count and a vaccine, and in the chapters on "Diseases of the Nervous System" and "Metabolism." The parts devoted to diseases of the lungs and tuberculosis are lamentably weak; no mention is made of the aspiration of fluid from the pleural cavity by means of oxygen replacement, and apparently artificial pneumothorax and heliotherapy are not practised in America. Hydrotherapeutic treatment is advocated in preference to the use of drugs as a means to reduce fever, whilst for patients suffering from typhoid the brand or full bath is recommended; indeed, there are many instances which indicate that American methods approximate more nearly to French than English standards. The book is admirably illustrated and contains details of treatment likely to be of interest to students of international medicine.

**"The Nursing Mirror" Pocket Encyclopædia and Diary, 1921.** (The Scientific Press, Ltd. 1s. 8d. net, post free.)

The 1921 issue of this wonderful compendium of information contains much new matter and fresh illustrations. Among the former is useful information on the "Classification of Bacteria." The illustrated section on "Decorations and Medals, for which Nurses are Eligible," now includes the Victoria Cross. The book which must prove a mine of useful information to nurses which they might not otherwise be able readily to get.

